# Spatiotemporal Impacts of Enceladus- and Earth-relevant Ammonia Gas On Cultivation of Extremophile *Halomonas meridiana*

**DOI:** 10.1007/s00248-025-02621-1

**Published:** 2025-10-20

**Authors:** Cassie M. Hopton, Charles S. Cockell

**Affiliations:** UK Centre for Astrobiology, School of Physics and Astronomy, University of Edinburgh, James Clerk Maxwell Building, Peter Guthrie Tait Road, Edinburgh, EH9 3FD UK

**Keywords:** Ammonia, Extremophiles, Habitability, Enceladus, Astrobiology, Pollution

## Abstract

One underexplored aspect of microbial growth is the impact of toxic gases transported through the atmosphere. Ammonia is a gas that can supply essential nitrogen but also exert cellular toxicity. Ammonia volatilized from a concentrated source into surrounding environments is therefore a crucial consideration when assessing the capacity of environments to support life, such as within terrestrial environments polluted with ammonia, or the ice crusts above ammonia–water oceans of icy moons. We cultivate *Halomonas meridiana* proximal to an ammonia source and examine the impact of ammonia volatilization on growth. Lower cell densities (OD_600_ = 0–1) occurred nearest the ammonia source. At 24 h, wells exhibiting an OD_600_ = 0–0.5 were evident when ammonia concentrations were ≥ 0.5 M. *H. meridiana* in proximity to 0 M, 0.1 M, 0.25 M, 0.5 M, and 1 M ammonia exhibited OD_600_ > 2 in 89.86%, 57.97%, 37.32%, 30.07%, and 18.48% of culture wells at 48 h, respectively. Alteration to growth kinetics and viability of *H. meridiana* cultivated adjacently to an ammonia source (“adjacently exposed”) were not as severe compared to direct culture in ammonia (“directly exposed”). Compared to control, adjacent exposure to 0.1 M ammonia exerted no significant detrimental effect on growth kinetics and enhanced cell density, but adjacent exposure to ≥ 0.5 M ammonia greatly extended lag time, doubling time, reduced cell density, and reduced viability. Ammonia volatilized from 0.1 M sources may thus minimally affect, if not improve, habitability, whereas environments exposed to ammonia volatilized from sources at ≥ 0.5 M could constrain habitability.

**Supplementary Information:**

The online version contains supplementary material available at 10.1007/s00248-025-02621-1.

## Introduction

The habitability of an environment is influenced by its physicochemical conditions, the accessibility of chemical elements, nutrients and energy, and, distinctly, the availability of liquid water. Equally, the habitability of an environment can become constrained or limited by the presence of detrimental environmental components [1, 2]. One class of detrimental compounds is volatile toxic gases, which can be transported through the atmosphere. One such gas is ammonia (hereafter, the term “ammonia” is utilized to denote the total concentration of unionized ammonia, NH_3_, and the ammonium ion, NH_4_^+^, in an environment). Ammonia is a bioavailable nitrogen source and metabolic input for many living organisms including bacteria [3–5]. However, the toxicity of ammonia has been demonstrated across a number of species, from bacteria to humans [6–9]. Notably, NH_3_ possesses certain properties (e.g., small size and uncharged) which facilitates passive permeation through biological membranes. As a weak proton (H^+^) acceptor, NH_3_ readily reacts with H^+^ to raise pH, disrupt proton motive force, and form NH_4_^+^ which impacts ionic balance [10–13]. The presence of NH_3_ gas in the environment is therefore an important consideration when assessing habitability.

In aqueous environments, NH_3_ exists in equilibrium with NH_4_^+^. The proportion of each species of ammonia is determined primarily by pH, but also temperature, pressure, and salinity [14–16]. Under standard Earth conditions (temperate and standard pressure) and pH exceeding 9.25, over half of ammonia is present as NH_3_ gas. NH_3_ is thus commonly released into the terrestrial atmosphere following application of ammonium fertilizer to alkaline soils. The process of release is known as volatilization—a phenomenon whereby ammonia transitions to the gas phase (NH_3_) and escapes into the atmosphere. Due to volatilization, long-range dispersion and deposition of NH_3_ has been well demonstrated [17–20]. Ammonia has been detected up to 3 km from a source pollution site and could be detected at a level of 5.1 µg/m^3^ in natural reserves [20]. Despite the relatively short atmospheric lifetime of NH_3_ which is in the order of hours to days [21–23], atmospheric transport can thus deposit ammonia into surrounding environments [24–26]. It is therefore possible for NH_3_ to disperse from a concentrated, local source to distant environments. Such deposition may impact the habitability of afflicted environments.

As one of the simplest organisms on Earth, bacteria can provide a valuable foundation to examine growth limitations when considering habitability. Growth limitations in ammonia have been established for *Bacillus subtilis* [27, 28], *Escherichia coli* [28], *B. pasteurii*, *B. pumilus* [27], sulfate-reducing bacteria [29], and the extremophile *Halomonas meridiana* [30]. Increasing concentrations of atmospheric NH_3_ has been found to reduce the number of viable bacteria [31]. NH_3_ gas dissolved into environments from a distant source could thus be presumed to elicit one of two distinct effects. At low concentrations, the deposition of dispersed NH_3_ could support the growth of organisms by providing essential nitrogen [32–36]. Alternatively, at high concentrations, NH_3_ could exert adverse effects on the biodiversity of aquatic life, plants, invertebrates, and bacteria [37–41]. However, this binary effect of NH_3_ has not been explicitly demonstrated in research. Additionally, NH_3_ dispersed in the atmosphere can presumably alter growth over a wider spatial range than direct deposition but may be less toxic due to diminished concentration. The effects of NH_3_ are also likely to be temporary as ammonia disperses into air over time. These facets of atmospheric NH_3_ gas generate questions, such as what this spatial effect on bacterial growth could look like, and whether bacteria could recover from NH_3_ gas exposure after complete dispersal. Such fundamentals are important when considering how terrestrial bacteria may be impacted by anthropogenic ammonia pollution.

Crucially, these fundamentals could also inform extraterrestrial habitability. Icy moons of our solar system feature liquid water subsurface oceans encased below thick ice shells [42–44]. The liquid state of the oceans has been thought to be preserved by anti-freeze components such as ammonia [45, 46]. Supporting this, mass spectrometry analysis by the Cassini–Huygens mission of the material ejected from the Southern plume of Saturn’s icy moon Enceladus—presumed to originate from the subsurface ocean—measured ammonia at a volume mixing ratio of 0.4–1.3% [47, 48]. Enceladus features an availability of heat [49, 50], energy [51–53], nutrients [47, 48, 54, 55], and organics [47, 56], as well as liquid water, essential for prebiotic chemistry. Enceladus is thus a prominent target in astrobiology and the search for habitable environments beyond Earth.

On Enceladus, the spatial and temporal impact of NH_3_ gas could be relevant to habitability prospects in the brine channels, veins, pockets, and fractures presumed to feature in the ice shell. These networks have been hypothesized as potentially habitable environments [57–60]. NH_3_ gas may permeate into these environments through multiple pathways. In one pathway, NH_3_ gas may be released and adsorbed onto the ice shell following exsolution from the ocean [61, 62] and migrate to the fluid networks by surface diffusion. Alternatively, ice fractures that are open to the surface may enable NH_3_ volatilization on icy moons. This would depend on local temperature (−23 to 0 °C or higher) and pH (greater than pH 9) [63], as well as pressure. NH_3_ can easily volatilize on Earth; the air contains trace amounts of ammonia, creating a strong gradient driving ammonia from environments to the air. On icy moons, NH_3_ escape is governed by Henry’s Law and vapor pressure equilibrium: colder temperatures reduce NH_3_ volatility, and thus near-zero pressures are needed for NH_3_ to escape, volatilize, from liquid. Such conditions could exist in ice shell fractures connected to the surface of Enceladus which is under near vacuum pressure. Volatilized NH_3_ could then migrate along the fracture system and potentially solubilise into intersecting brine networks within the ice shell. As with exsolved NH_3_, volatilized NH_3_ may also absorb onto ice walls of the fracture and enter brine networks through ice diffusion [62]. It is not without mention that other prominent astrobiology targets, the icy moons Titan, Europa, Callisto and Ganymede, are also modelled with an internal ocean of NH_3_ where spatial and temporal effects of NH_3_ gas could be relevant [42, 46, 64]. However, the presence of NH_3_ in these oceans has yet to be confirmed by direct measurements.

To characterize the habitability impacts exerted by volatilized and dispersed NH_3_ gas, this work investigates the cultivation of *Halomonas meridiana* (nomenclature synonym: *H. aquamarina*) circumjacent to NH_3_ gas. *H. meridiana* is a deep-sea bacterium with no specialized ammonia adaptations [65] but has extremophilic adaptations relevant to the physicochemical characteristics of the Enceladus ocean [66]. Indeed, we have previously shown *H. meridiana* can grow in concentrations of ammonia above the lower putative ammonia concentration threshold (0.01 M [61]) expected on Enceladus [30]. We utilize optical density readings to characterize the spatial impact of incrementally increasing concentrations of ammonia from 0.1 M to 1 M on growth. We analyze temporal recovery of cell density following exposure. Using growth kinetic and cell viability assays, we compare the growth kinetics of *H. meridiana* cultured adjacent to an ammonia source (“adjacently exposed”) to those directly exposed to ammonia (“directly exposed”). Through this, we assess implications for the habitability of environmental niches susceptible to NH_3_ gas exposure such as those on the icy moon Enceladus as well as Earth.

## Methods

### Bacterial Strain Selection and Culture

The gram-negative bacterium *Halomonas meridiana* Slfth1 (DSM 15724) was obtained from the German Collection of Microorganisms and Cell Cultures (DSMZ). *H. meridiana* remains validly published as a heterotypic synonym according to the International Code of Nomenclature of Prokaryotes (ICNP). However, it should be noted this strain has been synonymized with *H. aquamarina* based on phylogenomic classifications [67]. *H. meridiana* was isolated from low temperature hydrothermal fluid at a depth of 2000 m. As such, *H. meridiana* presents physiological traits necessary for growth in this environment. This includes halophilic adaptations (growth in up to 22% NaCl), alkalitolerance (tolerance up to pH 12), psychrotolerant traits (growth at −1 °C and cold shock protein genes), and piezotolerance (growth at 550 bar) [65, 66, 68]. Evidence of hydrothermal systems is present on Enceladus [48, 52]. The isolation location and physiological adaptations exhibited by this organism therefore make it a suitable model for establishing the limits of life on Earth and potential for habitability on icy moons where cold, saline, alkaline, and high-pressure waters would be expected. Additionally, the strain genome hosts no known ammonia adaptations [65] (DDBJ, accession no. AP022821). This was an intentional choice. The estimated ammonia concentrations within icy moons oceans are low and may preclude the need for distinct ammonia adaptations. As such, the intention of this study was not to assess an already established ammonia adaptation, but to assess whether NH_3_ gas acts as a chemical parameter that can affect habitability of organisms without ammonia adaptations. Pure cultures of *H. meridiana* were maintained in a simplistic yeast media consisting of 1 g/100 mL Bacto™ yeast extract (Becton, Dickinson and Company), 0.2 M NaCl (1.17% salinity) (Thermo Fisher Scientific, CAS Number: 7647-14-5), and distilled water (dH_2_O) at pH 6. Cultures were cultivated aerobically in conical Erlenmeyer flasks with orbital agitation at 150 RPM and 28 °C.

### Ammonia Preparation

All ammonia solutions were prepared from a stock solution of 35% liquid ammonia (Fisher Scientific, CAS Number: 1336-21-6). Stock ammonia was diluted in yeast media to molar concentrations of 0.1 M, 0.25 M, 0.5 M, and 1 M ammonia and maintained in air-tight falcon tubes to prevent gaseous escape. Control solutions of 0 M ammonia consisted of yeast media with no ammonia supplementation. The pH of all solutions was unaltered to preserve incrementally higher concentrations of NH_3_ gas. The pH of each solution was recorded as follows: control (0 M), pH 6.06 ± 0.07; 0.1 M, pH 9.73 ± 0.01; 0.25 M, pH 10.18 ± 0.03; 0.5 M, pH 10.49 ± 0.01; and 1 M, pH 10.78 ± 0.02. The pH of solutions was determined with a Jenway 3510 benchtop pH meter.

### Spatiotemporal Analysis of Ammonia Toxicity

Experiments were conducted in polystyrene 96-well plates. These plates were not air-tight and thus permitted ammonia to travel from wells to the exterior atmosphere. All wells, with exception of four central wells (D6, D7, E6, and E7), were plated with 190 µL yeast media inoculated with 10 µL overnight *H. meridiana* culture to an optical density at 600 nm (OD_600_) of 0.05. A blank plate was created identically but without inoculation. The four central wells (D6, D7, E6, and E7) were plated with 200 µL ammonia at concentrations of either 0 M (control), 0.1 M, 0.25 M, 0.5 M, or 1 M, prepared as described above. Plates were incubated at 28 °C in a table-top orbital shaker at 150 RPM with OD_600_ readings taken at 24 and 48 h using a BMG SPECTROstar Nano Microplate Reader. Plates were shaken at 200 RPM before each reading. All plates were blank corrected against the blank plate before reading. Plate lids were utilized during incubation and analysis to ensure planar spread of NH_3_ gas across the plate. The liquid culture depth was approximately 6 mm in a total well depth of 10.67 mm. An open space of 1 mm was between the well top and the plate lid.

### Growth Experiments of Direct and Adjacent Ammonia Exposure

**Fig. 1:**
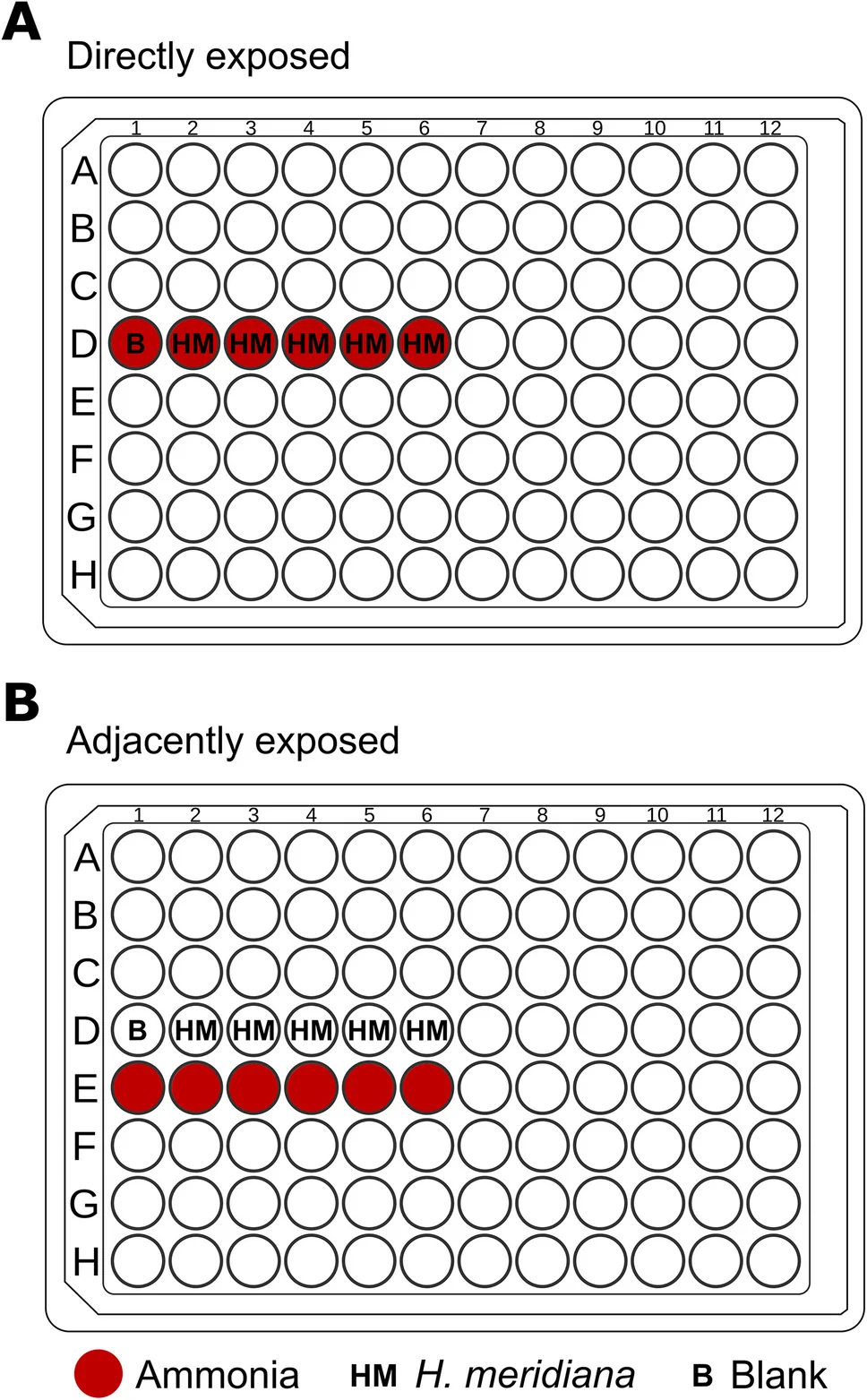
Diagram of “directly” and “adjacently” exposed cultures to ammonia. (**A**) Depiction of *H. meridiana* cultures directly exposed to ammonia (red circles). *H. meridiana* (HM) is cultivated in yeast media with ammonia to concentrations of 0.1 M, 0.25 M, 0.5 M, or 1 M ammonia. (**B**) Depiction of *H. meridiana* cultures adjacently exposed to ammonia. *H. meridiana* is cultivated in yeast media without ammonia. Ammonia at concentrations of 0.1 M, 0.25 M, 0.5 M, or 1 M were placed in the wells directly below these cultures. Blank (B) solutions are identical but are without inoculation of *H. meridiana*

Growth experiments were conducted within polystyrene 96-well plates utilizing two distinct culture conditions: direct and adjacent ammonia exposure. In the direct ammonia condition, ammonia concentrations of 0 M (control), 0.1 M, 0.25 M, 0.5 M, and 1 M in yeast media were prepared as outlined above and dispensed into five wells. Ammonia solutions were inoculated with 10 µL overnight culture of *H. meridiana* to OD_600_ = 0.05 in a final volume of 200 µL (Fig. 1A). In the adjacent ammonia condition, five wells of yeast media were inoculated with 10 µL overnight culture of *H. meridiana* to OD_600_ = 0.05 in a final volume of 200 µL. Five wells directly adjacent to these culture wells were supplemented with ammonia in yeast media to concentrations of 0 M (control), 0.1 M, 0.25 M, 0.5 M, or 1 M ammonia (Fig. 1B). In both growth conditions, separate 96-well plates were used for each ammonia concentration. Negative controls had no inoculation.

### Kinetic Growth Assays and Parameters

Following preparation of the growth experiments as outlined above, growth over time was assessed using OD_600_ readings over 48 h in a BMG SPECTROstar Nano Microplate Reader. Plates were shaken at 200 RPM before each reading. Growth parameters of lag phase duration, doubling time, and final OD_600_ at 48 h were extrapolated from the OD_600_ growth curve. Lag phase duration was determined using the online microbial lag phase calculator developed by Smug et al. [69]*.* The following parameters were applied: algorithm = parameter fitting to a model; preprocessing applied: cut data at some time = yes, max time = 24 or 48 h; smooth data = no; initial biomass = first observation; model to fit = logistic; NLS fitting algorithm = auto; and max number of iterations = 100. Doubling time was derived from the growth rate, *µ*, by the equation ln2/*μ*. Calculation of *µ* is presented in Eq. 1.0. *N*_0_ is the OD_600_ at a beginning time interval (*t*_0_) in the exponential growth phase. *N* is the OD_600_ at the end of a selected time interval (*t*) in the exponential growth phase. *t* and *t*_0_ were recorded in minutes.

1 $$\mu =\left({\mathrm{Log}}_{10}(N)-{\mathrm{Log}}_{10}({N}_{0})\right)2.303/(t-{t}_{0})$$

Final cell density was determined at 48 h using the final OD_600_ value obtained. We defined no growth as any analysis where there was no defined lag or exponential phase after 48 h. Increases to OD_600_ were confirmed to correspond to increases in cell viability (Supplementary Fig. S1).

### Cell Count

Cell viability of *H. meridiana* was assessed in cultures directly and adjacently exposed to ammonia. The experimental set-up of the two conditions occurred analogously to that described in the growth experiments. Then, 96-well plates were incubated for 4 h at 28 °C on a table-top orbital shaker at 150 RPM. This incubation period was found to be a suitable length of time to allow volatilization of NH_3_ from the central ammonia wells and complete dispersal across adjacent wells utilized for testing. The duration is also within the doubling time range of *H. meridiana* (i.e., 1 to 2 h). Following incubation, cell numbers were determined using colony forming units (CFU) on yeast media agar plates incubated at 28 °C.

### Evaluation of Ammonia Concentration

Volatilization and permeation of NH_3_ from and into cultures that were directly and adjacently exposed to ammonia was assessed by using the CHEMetrics High Range VACUette Ammonia test kit (K-1510C). The K-1510C kit features a detection range of 0–10,000 ppm ammonia and a detection limit of 100 ppm. Precision data is not available for this kit. However, a related kit, K-1513, has shown precision of ± 11 ppm at 112 ppm. This kit measures ammonia by a direct nesslerization reaction. Direct nesslerization determines ammonia concentration by a reaction of ammonia with potassium mercuric iodide. This produces a yellow-colored complex, the Nessler reaction product, which can be measured at 420 nm [70]. The experimental set-up of the two conditions occurred as described in the growth experiments. A 4-h incubation period was identified as a suitable length of time that could allow NH_3_ volatilization, dispersal, and dissolution into adjacent cultures. Plates were incubated for 4 h at 28 °C on a table-top orbital shaker at 150 RPM. The parts per million (ppm) of ammonia in each culture was determined by colorimetric analysis of the Nessler reaction product at 420 nm. Ammonia content in ppm was determined by linear regression; the absorbance of the culture sample at 420 nm was compared against a calibration curve created by absorbance of known concentrations of ammonia at 420 nm (Supplementary Fig. S2). Molarity (M) was derived from ppm as shown in Eq. 2.0. Where 1 ppm ≅ 1 mg/L, and molar mass refers to the molar mass of ammonia at 17.031 g/mol. 

2 $$M=\frac{(\mathrm{ppm}/1000 )}{{Molar \; mass}}$$

### Statistics and Reproducibility

All data were compiled from a minimum of three biological replicates (*n* = 3 to 4). The Shapiro–Wilk test was utilized to assess normality of data. Where the assumption of normality was violated, two groups were analyzed by the Mann–Whitney test or, for three or more groups, by the Kruskal–Wallis test. Multiple comparison correction was applied using Dunn’s test. Where normality was not violated, sample variance was assessed by an *F*-test for two groups. Samples of equal variance were analyzed by two-tailed unpaired *t*-test. If the assumption of variance was violated, a two-tailed unpaired *t*-test with Welch’s correction was applied. For three or more groups, the Brown–Forsythe test was utilized to assess equal variance. Samples of equal variance were analyzed by analysis of variance (ANOVA) followed by Tukey’s post hoc test. Samples of unequal variance were assessed by Welch’s ANOVA test with Tamhane’s T2 post hoc test. Data is presented as the mean ± standard deviation (SD). Significance was considered when *p* < 0.05. Statistical tests are specified in figure legends as well as the supplementary material when indicated. All figures and statistical analyses were produced using GraphPad Prism version 8.0.2 (GraphPad Software Inc.).

## Results

### Spatiotemporal Toxicity of Ammonia

**Fig. 2:**
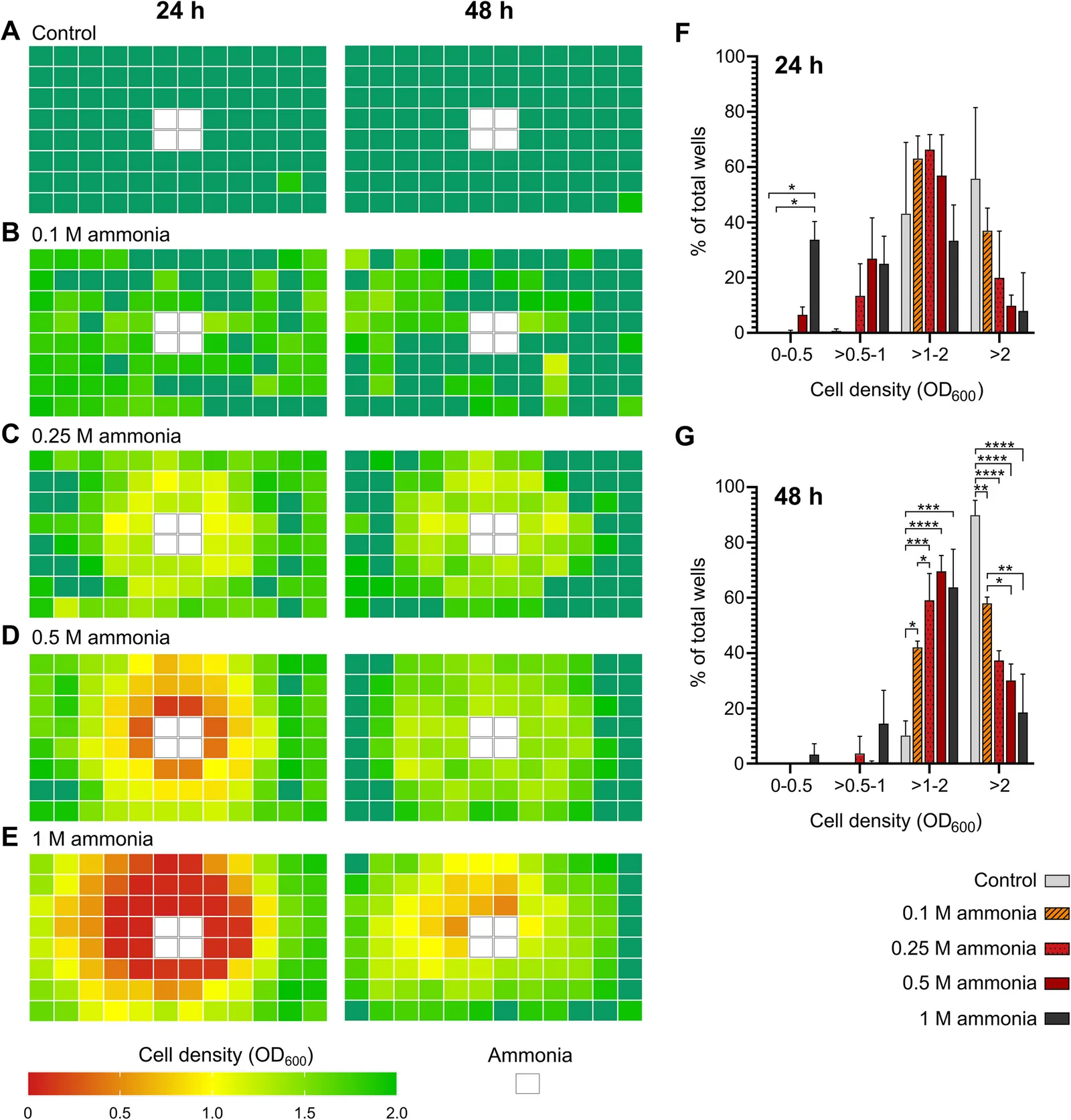
Spatiotemporal effect of ammonia on growth of *H. meridiana*. (**A–E**) Heatmaps representing the mean OD_600_ of wells in a 96-well plate (*n* = 3). Central wells (D6, D7, E6, and E7) were filled with either 0 M (control), 0.1 M, 0.25 M, 0.5 M, or 1 M ammonia as indicated by white squares. Surrounding wells were yeast media inoculated with *H. meridiana*. OD_600_ values were recorded 24 h and 48 h post-inoculation to ammonia. OD_600_ values were coloured as follows: 0, red; 1, yellow; 2, green; and  > 2, dark green. (**F, G**) Cell density changes with increasing ammonia concentrations. For each concentration of ammonia, the number of wells between OD_600_ 0–0.5, > 0.5–1, > 1–2, and > 2 at 24 h and 48 h post-inoculation was determined and calculated as a percentage of the total number of inoculated wells (92). The results are represented as a column graph with column heights and error bars indicating the mean ± S.D (*n* = 3). Statistical significance in (**F**) and (**G**) is given by the Kruskal–Wallis’s test using Dunn’s multiple comparison test for optical density groups 0–0.05 and > 0.5–1, and one-way ANOVA using Tukey’s multiple comparison test for optical density groups > 1–2 and > 2. ns, no significance; ^*^, *p* < 0.05; ^**^, *p* < 0.01; ^***^, *p* < 0.001; ^****^, *p* < 0.0001

NH_3_ is a gas under temperate conditions and standard pressure. The spatiotemporal impact of NH_3_ on bacterial cultures was assessed using 96-well plates. Plates were prepared with four central wells containing either yeast media (0 M ammonia, control) or ammonia at concentrations of 0.1 M, 0.25 M, 0.5 M, and 1 M. The peripheral wells were filled with yeast media inoculated with *H. meridiana*. Figure 2A-–2E is a heatmap illustrating the cell density of wells surrounding ammonia concentrations of 0 M (control, Fig. 2A), 0.1 M (Fig. 2B), 0.25 M (Fig. 2C), 0.5 M (Fig. 2D), and 1 M (Fig. 2E) at 24 h and 48 h post-inoculation. Cell density is given by optical density values at 600 nm (OD_600_). Values of cell density are characterized as follows: 0—red, 0.5—orange, 1—yellow, 1.5—green, and  ≥ 2—dark green. Figure 2F-G indicate the total number of wells, as a percentage of the total number of culture wells (92), that reached cell densities between 0–0.5, > 0.5–1, > 1–2, and > 2 OD_600_ at 24 h and 48 h, respectively, for each control and ammonia treatment condition.

Figure 2 demonstrates a pronounced suppression of cell density which was most severe in proximity to the ammonia source as ammonia concentration increases. Culture wells circumjacent to the control solution at 24 h and 48 h, with exception of a single well in each case, showed an average cell density at or exceeding 2 (Fig. 2A). At 24 h, 43.12% of culture wells in the control condition were at a cell density between OD_600_ > 1–2 and 55.8% of wells at a cell density OD_600_ ≥ 2 (Fig. 2F). At 48 h, 89.86% wells exhibited a cell density of OD_600_ ≥ 2 (Fig. 2G). The control represents typical growth densities that would be expected of *H. meridiana* when cultivated in optimal conditions. In culture wells circumjacent to 0.1 M ammonia, all wells with exception of one exceeded a cell density of OD_600_ = 1 (Fig. 2B). At 48 h, 57.97% of wells were OD_600_ ≥ 2. This was lower than the number of wells at or exceeding a cell density of OD_600_ ≥ 2 at 48 h in the control condition (*p* < 0.01), but higher than the number of wells at a cell density of OD_600_ ≥ 2 when compared against the 0.25 M (*p* < 0.05) and 0.5 M (*p* < 0.01) ammonia conditions at 48 h (Fig. 2G).

The majority of cultures wells circumjacent to ammonia at a concentration of 0.25 M exhibited a cell density between OD_600_ = 1 and OD_600_ = 2 at both 24 h and 48 h (Fig. 2C). In this culture system, 19.93% of wells exhibited cell density values of OD_600_ ≥ 2 at 24 h (Fig. 2F). This increased to 37.32% at 48 h (Fig. 2G). The number of wells at a cell density of OD_600_ ≥ 2 was found to be nonsignificantly different from the 0.1 M (*p* = 0.0664), 0.5 M (*p* = 0.808) and 1 M ammonia (*p* = 0.101) conditions but lower than the control condition at 48 h (*p* < 0.0001) (Fig. 2G).

In the 0.5 M ammonia condition, 9.78% of wells exhibited a cell density of OD_600_ ≥ 2 at 24 h (Fig. 2F). The number of wells at this level increased to 30.07% at 48 h but was still lower than that observed in control (*p* < 0.0001) and 0.1 M ammonia (*p* < 0.05) conditions (Fig. 2G). A cell density of OD_600_ < 0.5 at 24 was observed in 6.52% of wells (Fig. 2F), but none were observed at this level at 48 h (Fig. 2G). Comparatively, 7.97% of wells exhibited a cell density of OD_600_ ≥ 2 when surrounding 1 M ammonia at 24 h, while 33.7% of wells exhibited an OD_600_ < 0.5 (Fig. 2F). At 48 h, the number of wells at a cell density of OD_600_ ≥ 2 increased to 18.48%, but the majority of wells were between OD_600_ = 1–2 (63.77%) and 3.62% of wells were at a cell density of OD_600_ < 0.5 (Fig. 2G). Notably, there was no significant difference found between the mean percentage of wells between > 0.5–1, > 1–2, and > 2 OD_600_ compared across all treatment concentrations at 24 h, but treatment with 1 M ammonia significantly increased the number of wells between OD_600_ = 0–0.5 compared to control (*p* < 0.05) and 0.1 M conditions (*p* < 0.05) (Fig. 2F).

For *H. meridiana* cultivated proximal to 0.25 M, 0.5 M, and 1 M ammonia, lower cell density values (OD_600_ < 1) were nearer to the ammonia source at both 24 h and 48 h, while the horizontal and vertical perimeter wells showed higher cell densities (OD_600_ > 1) (Fig. 2C-–2E). In addition, the radial distribution of NH_3_ was reflected in cell density alterations; the spatial zone of alterations became larger as ammonia concentration increased.

**Fig. 3:**
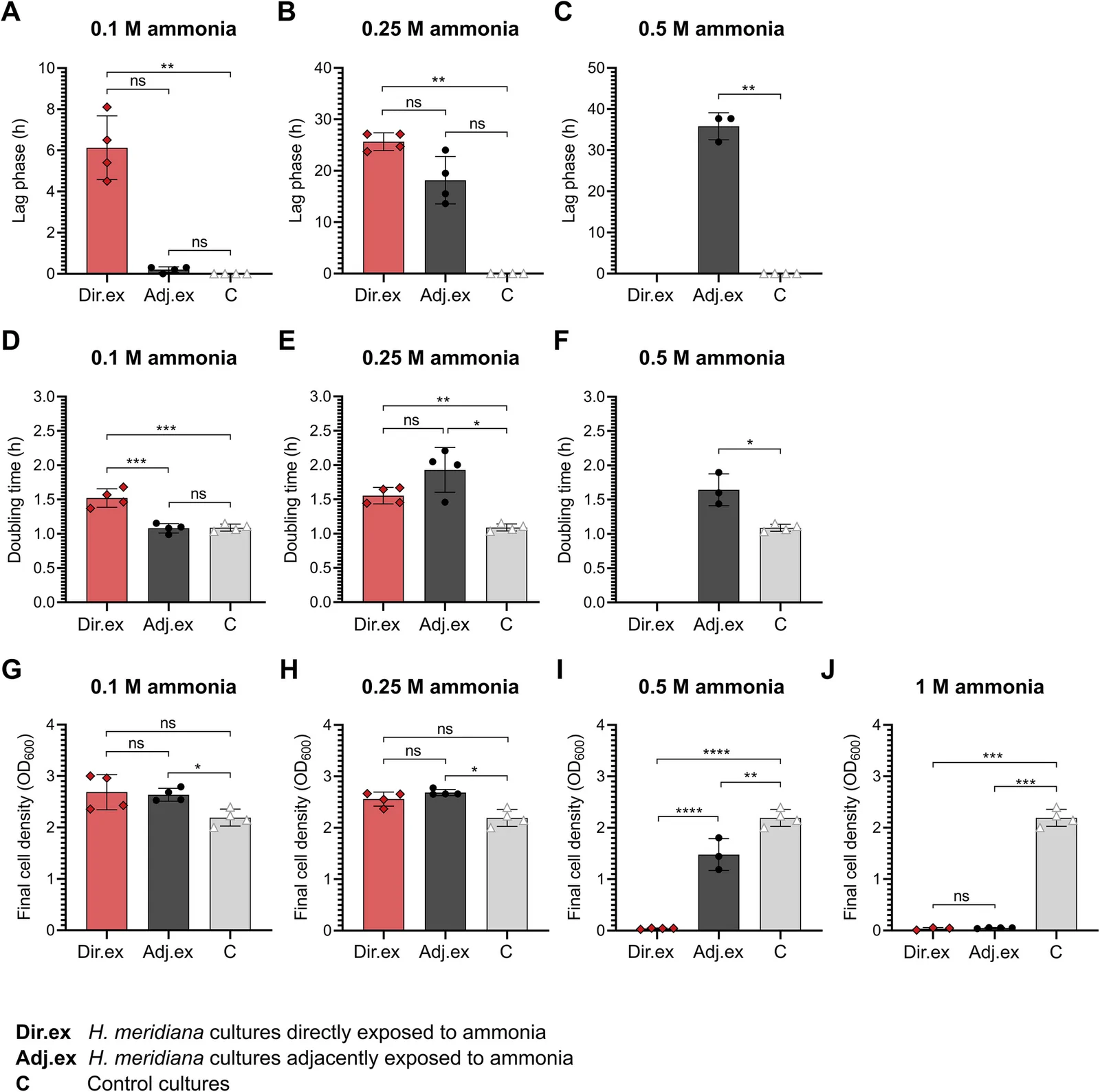
Growth kinetics of *H. meridiana* cultivated directly and adjacently to ammonia. (**A–C**) Lag phase time, (**D–F**) doubling time, and (**G–J**) final OD_600_ of *H. meridiana* at 48 h extrapolated from growth curves of Supplementary Fig. S3. Column heights indicate the mean ± S.D. (*n* = 3 to 4). Statistical significance was calculated by one-way ANOVA, Welch’s ANOVA, Kruskal–Wallis test and Welch’s unpaired *t*-test. The individual tests utilised are outlined in Supplementary Table S1. ns, no significance; ^*^, *p* < 0.05; ^**^, *p* < 0.01; ^***^, *p* < 0.001; ^****^, *p* < 0.0001

### Kinetics of *H. meridiana* Cultivated Directly and Adjacently to Ammonia

Wells immediately adjacent to the ammonia source showed a lower cell density than those farther from the ammonia source. The lower cell density suggests an alteration to growth kinetics. Given the small (9 mm) distance from the ammonia source, the impact to growth kinetics could be similarly observed in *H. meridiana* directly exposed to ammonia. To explore this possibility, the growth dynamics of *H. meridiana* directly exposed to ammonia and adjacently exposed to an ammonia source were investigated and lag phase duration (Fig. 3A-–3C), doubling time (Fig. 3D-–3F), and final cell density (OD_600_) at 48 h (Fig. 3G-–3J) extrapolated. The growth curves utilized to extrapolate these growth parameters is available in Supplementary Fig. S3. *H. meridiana* exhibited an extension to lag phase duration when directly cultivated in all concentrations of ammonia. Adjacent cultivation to ammonia increased the duration of the lag phase at concentrations of 0.25 M and 0.5 M ammonia, but not 0.1 M ammonia (Fig. 3A-–3D). *H. meridiana* cultivated adjacent to 0.1 M ammonia wells assumed a lag phase time of 0.2 h ± 0.141 (Fig. 3A). This was a shorter duration than those cultivated directly in 0.1 M ammonia (6.13 h ± 1.55). Differences between lag phase duration of *H. meridiana* cultivated adjacently to 0.1 M ammonia was found to be non-significant from cells cultivated directly in ammonia (*p* = 2.00) and in control conditions (*p* = 0.665). Similarly, *H. meridiana* cultivated adjacent to 0.25 M ammonia wells assumed a lag phase time of 18.15 h ± 4.62 (Fig. 3B). This was a shorter duration than those cultivated directly in 0.25 M ammonia (25.65 h ± 1.72). Differences between lag phase duration of *H. meridiana* cultivated adjacently to ammonia were found to be non-significant from cells directly exposed to ammonia (*p* = 0.485) and to control conditions (*p* = 0.267).

In Fig. 3D, the doubling time of cells cultivated adjacently to 0.1 M were comparable to growth in control conditions (*p* = 0.989) and faster than cells cultivated directly in 0.1 M ammonia (*p* < 0.001). Conversely, as ammonia concentration increased, cells adjacent to 0.25 M ammonia showed comparable doubling time to cells cultivated directly in 0.25 M ammonia (*p* = 0.271). This was longer when compared to control conditions (*p* < 0.05) (Fig. 3E). *H. meridiana* adjacently exposed to ammonia showed comparable final OD_600_ to cells cultivated directly in ammonia at concentrations of 0.1 M (*p* = 0.991) (Fig. 3G) and 0.25 M (*p* = 0.999) (Fig. 3H). Notably, the cell density at 48 h was significantly higher in cells cultivated adjacently to ammonia than those in control conditions at 0.1 M ammonia (*p* < 0.05) (Fig. 3G) and 0.25 M ammonia (*p* < 0.05) (Fig. 3H). *H. meridiana* did not show an observable lag, log or stationary phase when cultivated directly in 0.5 M ammonia but did show onset of growth at ~ 30 h post-inoculation when cultivated adjacently to a 0.5 M ammonia source (Supplementary Fig. S3). Consequently, cultures adjacent to 0.5 M ammonia exhibited a higher final cell density compared to cells cultivated directly in 0.5 M ammonia (*p* < 0.0001) (Fig. 3I). *H. meridiana* cultivated adjacently to 0.5 M were found to have a longer lag phase duration (*p* < 0.01) (Fig. 3C), slower doubling time (*p* < 0.05) (Fig. 3F) and lower final cell density (*p* < 0.01) (Fig. 3I) than *H. meridiana* in control conditions. No growth was recorded in *H. meridiana* cultivated directly in or adjacently to 1 M ammonia (Supplementary Fig. S3). OD_600_ at 48 h in both conditions thus remained at 0.05 (Fig. 3J).

### Cell Viability and Ammonia Content in *H. meridiana *Cultures Directly and Adjacently Exposed to Ammonia

**Fig. 4:**
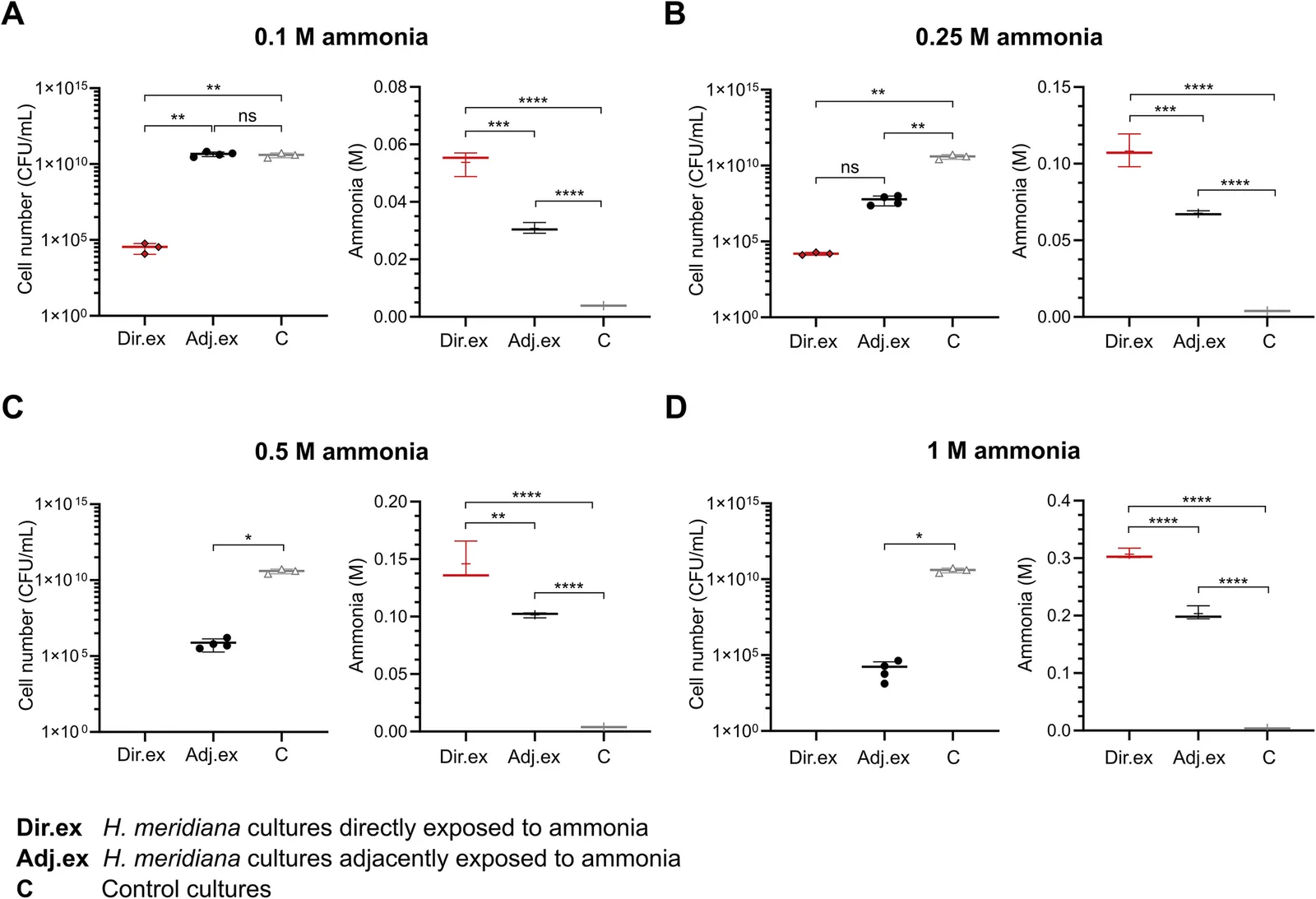
Cell number and ammonia content of *H. meridiana* cultures exposed to ammonia. Cell number and ammonia content at 4 h in *H. meridiana* cultures following direct and adjacent exposure to ammonia concentrations of 0.1 M (**A**), 0.25 M (**B**), 0.5 M (**C**), and 1 M (**D**) are shown. Comparison to cell number and ammonia content in 0 M (control) cultures for each concentration is illustrated. Cell number is given by CFU/mL in individual value scatter plots. Mean is indicated by the central line between individual points (*n* = 3). Error bars are ± S.D. Statistical significance was calculated by one-way ANOVA with Tukey’s post hoc test (0.1 M and 0.25 M) or two-tailed unpaired *t*-test with Welch’s correction (0.5 M and 1 M). Ammonia concentration is depicted in box plots that show molar concentrations of ammonia measured in culture solutions 4 h post direct and adjacent exposure to ammonia. Box plots represent the median as well as the 25% and 75% interquartile ranges. The whiskers represent 1.5 × the interquartile ranges. Plus sign (+) indicates the mean and the middle line indicate the median (*n* = 3). All statistical tests here correspond to one-way ANOVA with Tukey’s post hoc test. ns, no significance; ^*^, *p* < 0.05; ^**^, *p* < 0.01; ^***^, *p* < 0.001; ^****^, *p* < 0.0001

Lag phase duration of *H. meridiana* increased incrementally when cultivated adjacently to higher concentrations of ammonia. We have previously shown this extension to lag phase duration is a result of reduced viable cell populations correlating with ammonia concentration [30]. Figure 4 shows cell number of *H. meridiana* and ammonia content in cultures following 4 h of direct and adjacent exposure to ammonia. Control conditions without ammonia are also shown. Across all directly exposed ammonia cultures, the concentration of ammonia was diminished from original inoculation concentration at 4 h confirming ammonia volatilization from wells over time. Gaseous NH_3_ solubilized into adjacent cultures from an ammonia source was indicated by detectable levels of ammonia in these wells.

Differences in cell number were nonsignificant between control cultures and cultures adjacent to 0.1 M (*p* = 0.794) (Fig. 4A). This aligned with a nonsignificant change in lag phase between this condition and control conditions in Fig. 3A. The ammonia content in 0.1 M ammonia adjacently exposed cultures at 0.0308 M ± 0.0002, however, was significantly higher than those in the control condition (*p* < 0.0001). This indicates 0.1 M was not a concentration that detrimentally impacts cell number, and in turn, lag phase duration. Cell number was lower in cultures directly exposed to 0.1 M ammonia compared to adjacently exposed to ammonia (*p* < 0.01) and control conditions (*p* < 0.01). In correlation with this, ammonia content in directly exposed cultures at 0.0538 M ± 0.004 was higher than those in adjacently exposed ammonia cultures (*p* < 0.001) or control cultures (*p* < 0.0001) (Fig. 4A). This correlates with the results of Fig. 3A that demonstrate direct exposure of *H. meridiana* to 0.1 M ammonia increases lag phase duration compared with control.

Differences in cell number were significant between control cultures and cultures adjacent to 0.25 M ammonia *(p* < 0.01) (Fig. 4B). Cultures adjacently exposed to 0.25 M showed a concentration of ammonia at 0.0677 M ± 0.000 which was significantly higher than control cultures (*p* < 0.0001). This level of ammonia may account for cell number reduction in ammonia-adjacent cultures. However, the level of viable cell loss was not found to impact lag phase duration significantly between ammonia-adjacent and control cultures, as indicated in Fig. 3B. As a result of viable cell loss, cell number changes in cultures adjacently exposed to 0.25 M were found to be non-significant from cultures directly exposed to 0.25 M (*p* = 0.999) (Fig. 4B). The non-significant change in cell number between directly and adjacently exposed cultures did not correlate with ammonia. Cultures adjacently exposed to 0.25 M showed a lower ammonia concentration than cultures directly exposed to 0.25 M, which were found to be 0.108 M ± 0.01 (*p* < 0.001). These results indicate, while not as cytotoxic as direct ammonia exposure, adjacent exposure to 0.25 M ammonia demonstrates detrimental effects on viability. This may account for the longer doubling time observed in cultures adjacently exposed to 0.25 M (Fig. 3E) which was not observable in cultures adjacently exposed to 0.1 M (Fig. 3D).

There were no viable *H. meridiana* cultivated directly in 0.5 M (Fig. 4C) and 1 M ammonia (Fig. 4D). These solutions also demonstrated the highest ammonia content compared to adjacent and control cultures, with ammonia concentrations of 0.146 M ± 0.02 and 0.307 M ± 0.009, respectively. These concentrations were thus deleterious to *H. meridiana.* There were viable cells in cultures adjacent to 0.5 M and 1 M ammonia. This indicated adjacent cultures did not acquire ammonia to the concentrations observed in direct cultures at any point. Indeed, the ammonia content of ammonia-adjacent cultures was significantly lower than those in directly exposed cultures at 0.5 M (0.102 M ± 0.002, *p* < 0.01) and 1 M ammonia (0.203 M ± 0.01, *p* < 0.0001). Compared to control, the cell number in cultures adjacently exposed to 0.5 M (*p* < 0.05) (Fig. 4C) and 1 M (*p* < 0.05) (Fig. 4D) were lower. In accordance with this, the ammonia content was found to be significantly higher in cultures adjacent to 0.5 M (*p* < 0.0001) and 1 M ammonia (*p* < 0.0001) solutions compared to control.

## Discussion

The aim of this work was to characterize the spatiotemporal effect of NH_3_ gas on the cultivation of an extremophile, *H. meridiana.* Although the habitability of extraterrestrial environments is speculative, ammonia is known to be a component of extraterrestrial oceans [46, 47, 71]. Thus, our findings have relevance to the potential for habitability in these environments. Additionally, NH_3_ volatilization, resulting from agricultural and industrial pollution [72, 73], can deposit ammonia at great distances and may alter habitability in environments on Earth beyond the local site of release [17–20]. These results therefore advance the current understanding on how the gaseous state of NH_3_ may permeate into environments and impact the potential for a habitat to become or remain inhabited.

Although previous studies have investigated the effects of toxic gases on microbial growth, none of these studies have examined the spatial effects of gas on microbial growth. In literature, the growth of a diverse range of bacteria has shown to be disturbed most closely to a pollutant source. For example, as in the case of soils nearby tailing ponds of seepage pollution [74] or soils with crude oil contamination [75]. In accordance with this, we observed reduction of cell density nearest to an ammonia source. We also identified that increasing concentrations of NH_3_ gas increased the spatial size of the zone affecting cell density. The effects we observed are analogous to diffusion-limited effects observed in agar plates. An increasing zone of inhibition with increasing concentration of a toxic substance has been demonstrated in agar diffusion assays using non-gas substances such as propolis extract [76], lauric acid-KOH [77], chromium [78], and a range of organics, inorganics and organometallics [79]. Likewise, an increasing spatial zone of antibacterial activity has been observed for application of increasing concentrations of reactive oxygen species on *Pseudomonas aeruginosa* [80] and *E. coli* [81]. Our findings show how microbial growth can also be influenced by compounds, such as NH_3_, traversing through the atmosphere as well as through liquid media.

A notable alteration was that *H. meridiana* cultivated adjacently to ammonia at concentrations of 0.1 M and 0.25 M were able to establish a higher density at 48 h compared to control conditions. It is possible these concentrations were low enough, particularly following evaporation, that diffusion of NH_3_ across the cytoplasmic membrane into the cell in equilibrium with NH_4_^+^ were sufficient to promote growth by providing additional nitrogen [82]. NH_4_^+^ is assimilated by either the glutamine synthetase-glutamate synthase (GS-GOGAT) or glutamate dehydrogenase (GDH) pathway. In enzymology, *K*_m_ is the substrate concentration at which an enzyme’s reaction is half of its maximal velocity, *V*_max_. *K*_m_ values for GS, GOGAT, and GDH have been reported at 0.033 M [83, 84], 0.0002 M [85], and 0.029 M [86] in bacteria. At 4 h, the ammonia concentration in wells adjacent to 0.1 M were 0.0308 M ammonia, and adjacent to 0.25 M ammonia were 0.0678 M ammonia. These concentrations are thus suitable for GS and GDH to achieve 50% velocity or higher according to Michaelis–Menten kinetics and may account for enhanced cell density compared to control.

In *Halomonas*, ammonia nitrogen removal, and thus assimilation, rates have been reported at 5 mg N L^−1^ h^−1^ in the species *H. salifodinae* [87], 9.10 mg N L^−1^ h^−1^ for strain HN2 [88] and 22 mg N L^−1^ h^−1^ in strain B01 [89]. This approximately corresponds to the respective removal, and possible assimilation, of NH_4_^+^ at ≈ 0.00036 M h^−1^, ≈ 0.00064 M h^−1^, and ≈ 0.0016 M h^−1^. As aforementioned, cultures adjacent to 0.1 M and 0.25 M ammonia sources were found to accumulate ammonia at 4 h to concentrations of 0.0308 M and 0.0677 M, respectively. This indicates the ammonia provided into the wells were near 20-fold and 42-fold higher than the concentration utilised in assimilation per hour. Such abundance may have stimulated higher growth than the control which featured limited amounts of ammonia.

Such abundance of ammonia could also be deleterious to growth, however. While enhanced cell density was observed in cultures adjacent to 0.25 M ammonia, lag phase duration was higher, doubling time was slower and cell viability also lower compared to control. In contrast to 0.1 M ammonia, cultures adjacent to 0.25 M showed ammonia values that were substantially higher than the *K*_m_ values required for 50% *V*_max_ in nitrogen assimilation enzymes and typical rates of nitrogen assimilation. Ammonia at 0.25 M ammonia thus reflects a transition concentration whereby excess ammonia becomes deleterious to growth. It is notable adjacent exposure to NH_3_ gas at a concentration of  ≥ 0.5 M is the limit at which the following effects were observed: (i) reduced cell density surrounding the ammonia source (OD_600_ < 0.5); (ii) increased lag phase duration and doubling time; and (iii) reduced cell number of *H. meridiana* from control conditions. Cultures adjacent to 0.5 and 1 M solutions may have experienced enhanced nitrogen assimilation but likely experienced an overriding toxicity effect including internal alkalization and disruption to the internal H^+^ pool that ultimately led to the deleterious growth effects described. This is a function of ammonia concentration; we have previously demonstrated 0.05 M ammonia as a habitable limit for *H. meridiana* [30]*.* It is thus in accordance with this that cultures adjacent to 0.1 M ammonia showed an ammonia content below this limit. Concentrations above this limit were observed in 0.25 M, 0.5 M, and 1 M cultures where growth or viability was significantly impacted. Our findings indicating a correlation between increased concentrations of NH_3_ gas and reduced cell growth has also been demonstrated using other forms of toxic gas [90–96].

In extraterrestrial icy worlds, ammonia is a primordial component incorporated into subsurface oceans. Fluid networks are expected within the overlying ice shells and could function as potential habitats [57–60]. It is possible fluids within the ice shell networks closely mimic, or are identical to, the composition of the ocean below and thus contain ammonia. However, it is feasible NH_3_ gas may also be formed in these environments and thus influence habitability in addition to solubilised ammonia. For example, NH_3_ gas may be produced by a process of exsolution at the plume vent region of Enceladus and expelled. NH_3_ gas adsorbed onto the plume vent ice walls may then migrate through the ice shell to fluid networks. Alternatively, fractures through the ice shell that connect to the near vacuum surface could provide the pressure to facilitate NH_3_ volatilization from connected fluid networks or the ocean if also permitted by pH and temperature. Volatilized NH_3_ migrating up a fracture shaft may then disperse and dissolve into other fluid networks that intersect with the fracture region, impacting the potential for habitability in these environments.

The work presented here mimics an NH_3_ exposure scenario. We have shown that the spatial migration of ammonia impacts the concentration of ammonia dissolved into a solution and thus the growth of *H. meridiana*. We provide proof-of-principle evidence that the concentration of ammonia solubilized determines whether growth is facilitated or hindered. At low concentrations, addition of bioavailable nitrogen in the form of ammonia can facilitate growth, while higher concentrations can exert toxic cellular effects. On Enceladus, a hypothetical scenario can be argued; NH_3_ volatilized at the base of a fracture site may be more harmful at this site and less harmful as the gas migrates up, absorbs onto ice walls and fluid networks, and deceases in concentration.

We also demonstrated that environments neighbouring an ammonia concentration of 0.1 M improved growth of *H. meridiana*, while environments adjacent to concentrations exceeding 0.1 M became detrimental to growth and viability. This would indicate environments adjacent to such concentrations may be habitable but display an altered bacterial community structure. For example, alkaliphilic bacteria may survive and propagate with greater abundance than alkalitolerant bacteria, such as *H. meridiana*, that may grow slower*.* It is notable 0.1 M ammonia is the upper concentration boundary approximated for the ocean of Enceladus [61]. These thresholds are specific to *H. meridiana;* generalized habitability thresholds in NH_3_ gas could be further constrained using a diversity of bacterial species. Indeed, comparisons with literature show that direct exposure to 0.1 M ammonia has been found toxic to neutrophilic *E. coli* and *B. subtilis* under icy moon conditions [28]. In accordance with our results, solutions where the NH_3_ concentration exceeds 0.1 M can be survived by a diverse array of bacteria under standard terrestrial conditions including: *B. subtilis* T5 (0.15 M NH_3_) [27], isolated sulfate-reducing bacteria (0.2 M NH_3_) [29], *Staphylococcus aureus* (0.3 M NH_3_), *Enterococcus faecium* (0.3 M NH_3_), *Micrococcus luteus* (0.3 M NH_3_), *Salmonella typhimurium* (0.3 M NH_3_), *B. cereus* T41 (0.3 M NH_3_), *Proteus pumilus* (0.5 M NH_3_), *B. pumilus* (> 0.5 M NH_3_), and *B. pasteurii* (> 0.5 M NH_3_) [27]. Thus, there is reason to believe such concentrations of NH_3_ gas volatilized from an ammonia source could be survived by a range of species, although growth may not be optimal.

The temporal effects of NH_3_ gas were also demonstrated. Due to the use of an open-air system, cultures of *H. meridiana* exhibited cell density recovery with time when adjacently exposed to ammonia at all concentrations. This is comparable to our previous study which demonstrated *H. meridiana* cell numbers recovered over time with ammonia evaporation from culture media [30]. The spatial toxicity imposed by ammonia is therefore transient and suggests ecosystems exposed to NH_3_ would only be temporarily affected if there is a pathway to ammonia dispersal into the bulk atmosphere. This may occur on Enceladus if NH_3_ outgassing from the ocean is infrequent. Presumably, continuous exposure would exert a continuous deleterious effect on growth. It is crucial to note these results do not infer an increased or decreased likelihood of life existing on icy moons. Indeed, many other factors will influence habitability. It may be found these environments are habitable but not inhabited. Rather, these results show that NH_3_ gas is a chemical parameter that could influence the potential for habitability in a spatiotemporal manner. The presence of NH_3_ gas should thus be considered when constraining targets for astrobiological exploration.

On Earth, localised “point sources” of ammonia pollution have been identified in both agriculture and industry [72]. This would suggest long-term spatial effects on nearby bacterial communities according to the results of this study. *H. meridiana* was selected as an organism that could survive in cold, saline and alkaline fluids under pressure. However, *H. meridiana* is also a mesophile, grows optimally at standard pressure and can grow at neutral pH. This organism, and the growth results presented, can thus represent bacteria without extremophilic adaptations and indeed our results are in accordance with other, diverse bacteria as outlined.

To more accurately assess the ecological impact of NH_3_ gas, a natural extension of this work would be to consider evaporation rate of NH_3_. The importance of evaporation rate in temporal toxicity may account for a discrepancy observed between the cell density achieved at 48 h in cultures surrounding 1 M ammonia (Fig. 2E) compared to the cell density reached at 48 h in cultures adjacent to 1 M ammonia in Fig. 3J. The evaporation rate of essential oils has been shown to effect the minimal inhibitory dose (MID) against *Staphylococcus aureus* and *E. coli*, with rapid evaporation producing a higher vapour concentration that reduces the MID required [97]. This research indicates discrepancies between experiments could be attributed to altered rate of ammonia evaporation, and thus delivery of NH_3_, driven by differences in the culture instrumentation and shaking speed. A continuation of this work would be to alter atmospheric pressure or incorporate an air flow system. The incorporation of such measures could more accurately replicate in situ environments relevant to icy worlds and in anthropogenic NH_3_ pollution.

## Supplementary Information

Below is the link to the electronic supplementary material.

ESM1 (XLSX 73.0 KB)

ESM2 (PDF 507 KB)

## Data Availability

The raw data supporting the conclusions of this article are available in the supplementary dataset unless specified as part of the supplementary material.
