# Supplementary material for: Spatiotemporal Impacts of Enceladus- and Earth-relevant Ammonia Gas On Cultivation of Extremophile Halomonas meridiana
Source: Microb Ecol. 2025 Oct 20;88(1):111. doi: 10.1007/s00248-025-02621-1 (PMC12537597; doi:10.1007/s00248-025-02621-1)
Supplement: Supplementary file 2 — (PDF 507 KB) [file 248_2025_2621_MOESM2_ESM.pdf]

# Supplementary Material

*Microbial Ecology*

# Spatiotemporal impacts of Enceladus- and Earth-relevant ammonia gas on cultivation of extremophile *Halomonas meridiana*

Cassie M. Hopton[1] and Charles S. Cockell[1]

[1] UK Centre for Astrobiology, School of Physics and Astronomy, University of Edinburgh, James Clerk Maxwell Building, Peter Guthrie Tait Road, Edinburgh, EH9 3FD, United Kingdom

**Supplementary Figure S1. Calibration curve of optical density readings at 600 nm coupled with cell viability.** Column graph depicts optical density readings at 600 nm ($OD_{600}$) (left axis) and cell viability measured using the PrestoBlue™ cell viability assay (right axis) of *H. meridiana* over five time points within 24 h. Column heights present the mean ± s.d. ($n$ = 3).

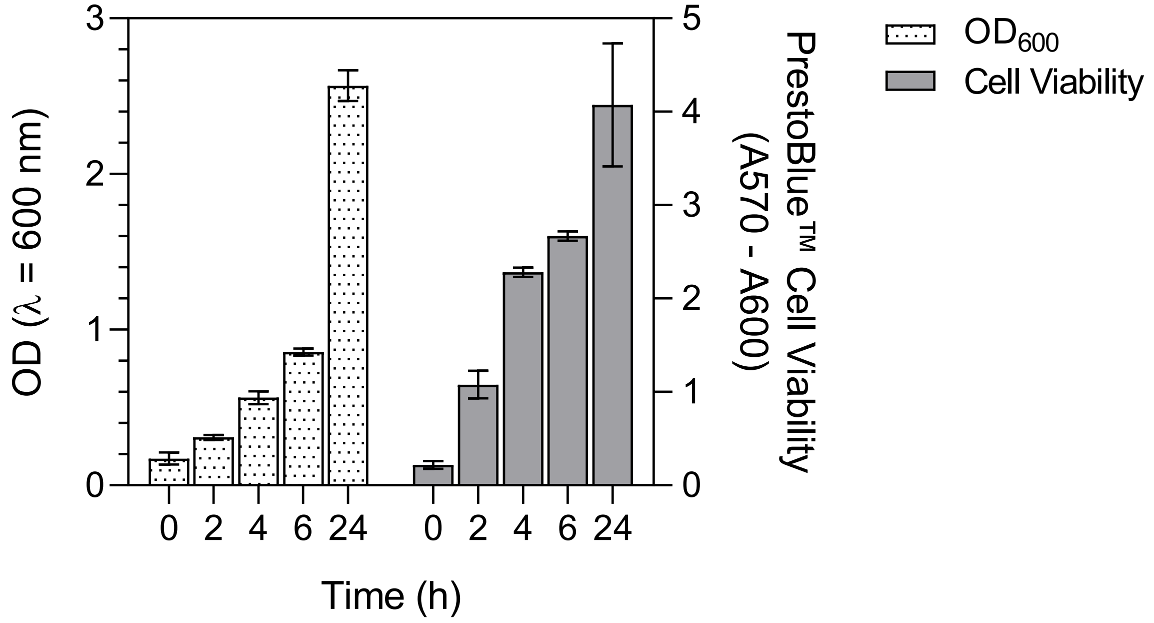

**Supplementary Figure S2. Direct nesslerization calibration curve.** Linear regression was created by measurement of known ammonia concentrations at 420 nm. Standard solutions of ammonia at known concentrations were provided by the CHEMetrics High Range VACUette Ammonia test kit (K-1510C). Linear regression equation utilised to calculate an unknown concentration of ammonia in ppm, X, is presented.

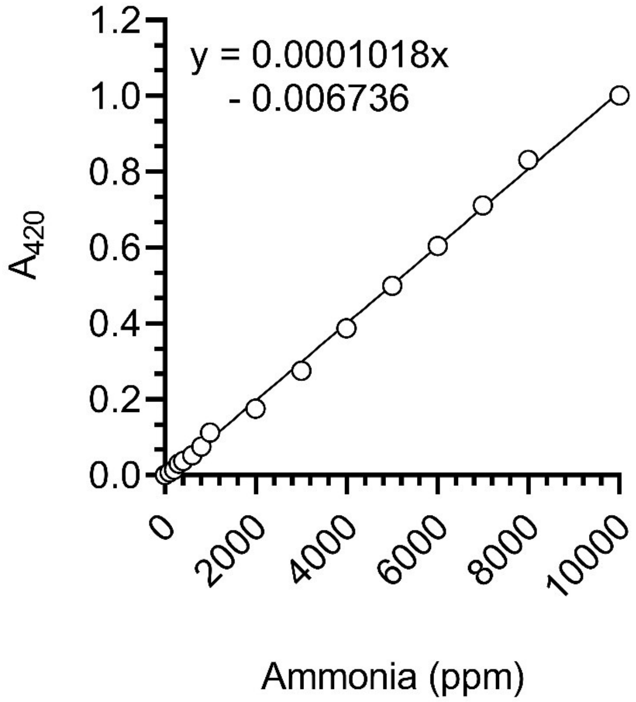

**Supplementary Figure S3. Growth dynamics of H. meridiana directly and adjacently exposed to ammonia.** Growth curves indicating optical density at 600 nm ($OD_{600}$) changes over time in *H. meridiana* directly exposed to and adjacently exposed to ammonia concentrations of (A) 0.1 M, (B) 0.25 M, (C) 0.5 M and (D) 1 M. All ammonia exposed cultures were compared to against growth of the control culture (0 M ammonia). Graph depicts mean ± S.D., with error indicated by area fill along error bands (*n* = 3).

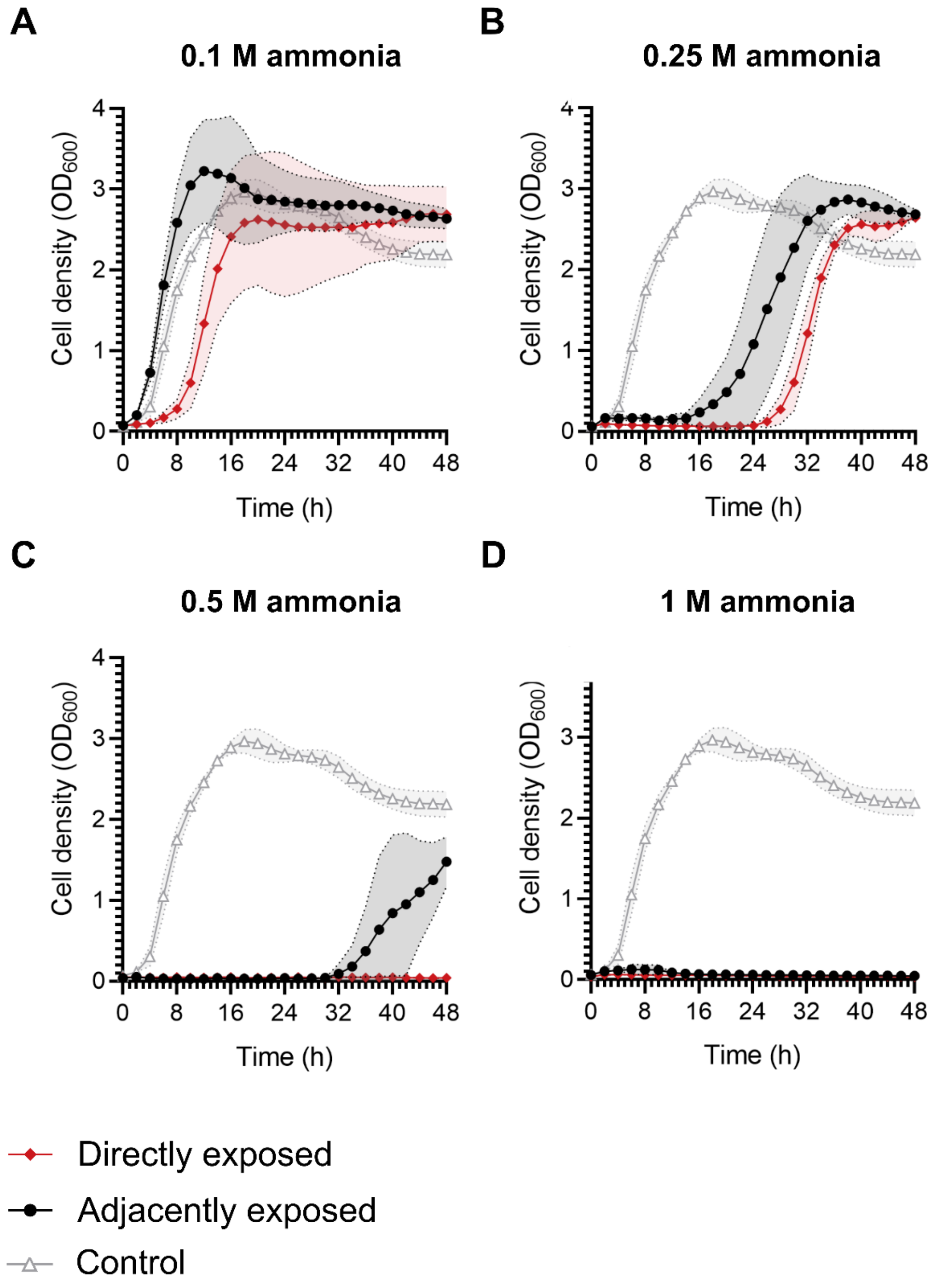

**Supplementary Table S1.** Statistical tests utilised in Figure 5 to compare mean lag phase, doubling time and Final $OD_{600}$ of *H. meridiana* grown in control conditions (0 M ammonia), and directly and adjacently to ammonia solutions of 0.1 M, 0.25 M, 0.5 M and 1 M.

| | **Lag phase** | **Doubling time** | **Final $OD_{600}$** |
|---|---|---|---|
| **0.1 M** | Kruskal-Wallis test, Dunn's multiple comparison test | One-way ANOVA, Tukey's multiple comparison test | Welch's ANOVA, Tamhane's T2 multiple comparison test |
| **0.25 M** | Kruskal-Wallis test, Dunn's multiple comparison test | Welch's ANOVA, Tamhane's T2 multiple comparison test | Kruskal-Wallis test, Dunn's multiple comparison test |
| **0.5 M** | Two-tailed unpaired t-test with Welch's correction | Two-tailed unpaired t-test with Welch's correction | One-way ANOVA, Tukey's multiple comparison test |
| **1 M** | n/a | n/a | Welch's ANOVA, Tamhane's T2 multiple comparison test |
